# Computational Creativity and Aesthetics with Algorithmic Information Theory †

**DOI:** 10.3390/e23121654

**Published:** 2021-12-08

**Authors:** Tiasa Mondol, Daniel G. Brown

**Affiliations:** 1 Untether AI, Toronto, ON M5V 2H2, Canada; 2 David R. Cheriton School of Computer Science, University of Waterloo, Waterloo, ON N2L 3G1, Canada

**Keywords:** computational creativity, computational aesthetics, algorithmic information theory, Kolmogorov complexity, typicality novelty and value, computational complexity

## Abstract

We build an analysis based on the Algorithmic Information Theory of computational creativity and extend it to revisit computational aesthetics, thereby, improving on the existing efforts of its formulation. We discuss *Kolmogorov complexity*, *models* and *randomness deficiency* (which is a measure of how much a model falls short of capturing the regularities in an artifact) and show that the notions of *typicality* and *novelty* of a creative artifact follow naturally from such definitions. Other exciting formalizations of aesthetic measures include *logical depth* and *sophistication* with which we can define, respectively, the *value* and creator’s artistry present in a creative work. We then look at some related research that combines information theory and creativity and analyze them with the algorithmic tools that we develop throughout the paper. Finally, we assemble the ideas and their algorithmic counterparts to complete an algorithmic information theoretic recipe for computational creativity and aesthetics.

## 1. Introduction

The principal idea in Algorithmic Information theory (AIT) is that we can quantify an absolute information content in an object with an algorithm or a program (a set of instructions) that gives rise to an object on some fixed machine. Named after the researchers who individually discovered this concept, such information complexity is known as the “Solomonoff–Kolmogorov–Chaitin” [1,2,3] or simply Kolmogorov complexity. Its classical counterpart, Shannon’s information theory, defines the entropy of an object as the length of code needed to encode it when it is a probabilistic outcome of a source (the more probable the object is as an outcome, the less entropy it has).

Such analysis is vital for when we talk about the communication overhead of a message, but it often is insufficient when we start talking about the essence of an individual entity. For example, the argument that the meaningful information content in “War and Peace” can be measured by including it in a set of possible novels with a probability distribution defined over the members, is a weak one [4].

This problem is less severe and more natural to analyze in an algorithmic setting, where a concise description of “War and Peace” would mean the description or algorithm “knows” about its global theme and at times Tolstoy’s intent. Moreover, such computation-focused method opens up ways of examining the object that goes beyond just measuring its information content: the length of the computation may point toward how information becomes buried under redundancy.

Additionally, the resources taken up by this process denote the difficulty involved in generating the object, and, perhaps most importantly, the corresponding algorithm helps measure the regularities and randomness embedded in the object.

We apply these information theoretic notions to computational creativity and aesthetics. Our work is made more challenging because of the difficulty in giving a holistic definition of creativity and of the aesthetic beauty of an artifact, but several criteria exist for its assessment, which have links to information measure. For example: Ritchie [5] proposes that typicality or novelty and value should be included in assessment of a creative artifact. The 4’P’ perspective of creativity [6] identifies the producer, process, press (context) along with the product as important dimensions of creativity and describes how each of these components is needed for the proper assessment of a creative artifact.

Formalizing these entities independently of each other is challenging; however, with the tools from AIT, we can investigate how these creative components may be identified when our reference point is the creative object itself. We motivate this by noting that the object is the first piece of tangible evidence that a creative phenomenon occurred. Additionally, the creator and creative process leave their identifying marks in the object, which later influences the perception invoked by the object in its observers.

In AIT, this is analogous to finding the *sophistication* (the aggregate of the creator’s stylistic properties in the object), *logical depth* (the computational difficulty associated with the generation of the artifact) and a minimum sufficient *model* (the regularities in the object as seen through a set of similar objects) of the artifact. Measures like this provide a firm computational underpinning of some fundamental yardsticks of creativity, and the focus of this paper is to look at a creative artifact through an algorithmic lens—taking each of the algorithm’s length, runtime, plausibility and meaningfulness into account.

### 1.1. Motivation

Application of algorithmic information theory in analysis of creative objects has been known for a while, but only the basic ideas have been explored. For example the plain and conditional version of Kolmogorov complexity [7], the latter of which denotes the information content with access to auxiliary data, have been shown to be remarkably effective in classifying and clustering symbolic music [8,9] into genres and composers. A broader scope of using Kolmogorov complexity in finding structure is universally applicable [10,11], but it is not always the case that we will find an object with low Kolmogorov complexity interesting or beautiful [12].

**Figure 1:**
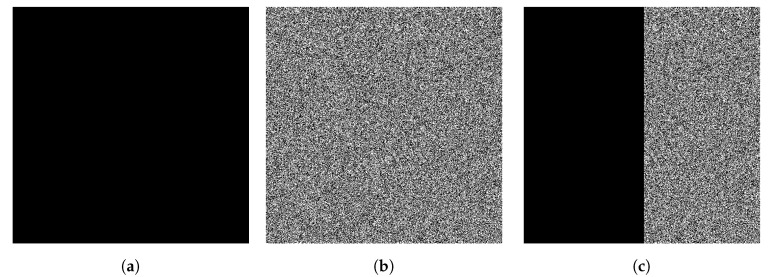
(**a**) is a simple highly compressible black canvas as compared to the random noise in (**b**). Neither has any aesthetic value. However, their combination (**c**) is also not valuable, illustrating the notion that raw complexity is not a measure of aesthetic quality: meaningless objects can be found across the spectrum of possible complexity scores. Image taken from: Zenil *et al*. [12].

Similarly, objects with high complexity can either be only random noise, which has the maximum possible Kolmogorov complexity or, in special cases, can store incompressible meaningful styles of its creator [13]; simply knowing the complexities of the objects is not enough to differentiate between these choices. Even looking for objects of “medium” complexity is insufficient: such an object might either be the product of substantial computational effort (which, in our telling, makes it a high-quality work) or could be trivial repeated patterns augmented with random bits to make it appear serious (Figure 1).

Only by looking at the actual computational effort undertaken by the object’s most probable source, that is, focusing on the creator, can we assess the object’s quality; the raw value of Kolmogorov complexity is insufficient. Additionally, while the conditional Kolmogorov complexity is an important measure of similarity between two objects [9], it is more useful to extract the regularities in an object that we shall call a *model* and then analyze the left-out conditional randomness. Then, a good *model* will be most successful in compressing the object and classifying it into a category that is most appropriate.

### 1.2. Summary of Results

In this paper, we build this theory of creativity of computationally created objects and expand on AIT driven ways to assess them. We begin by discussing preliminary definitions of Kolmogorov complexity and analyzing the *algorithmic probability* of an artifact, which is an important benchmark for assigning credibility to a given description of an artifact. In particular, the algorithmic probability tells us exactly why concision is preferred to redundancy, formalizing the Occam’s Razor principle. We then focus on the concept of *models*.

They describe the recognizable information in an artifact where we use a two-part code for its description; the other part describes the artifact’s individual aspects. The *model* describes what we normally associate with a genre or a creator’s oeuvre. We then show how *typicality* or *novelty* can be assessed for an artifact with respect to a model.

The concept of *logical depth* allows us to assess the computational effort required to produce an object: if an object with high compressibility has only slow-running short programs, the most likely explanation for the object is that it required substantial effort on the creator’s part to produce it, and it is valuable. Most remarkably, the concept of *sophistication* allows us to identify the signatory styles of a creator that can be used to generate objects that share these properties.

Highly sophisticated objects can be regarded as *masterpieces*, as they contain all the incompressible but non-random creative styles of a skilled creator. These concepts have not previously been extended to the domain of computational creativity. Next, after our full exploration of new ideas, we discuss some related works and revisit existing formulations of order and complexity to define computational aesthetics. A casual reader may skip ahead to this section for a more ready discussion on the related research on the current topic.

However, due to their dependence on the ideas presented hereafter, the reader can also benefit from reading through the paper sequentially. At the end of the paper, we summarize the ideas presented, with a complete recipe based on algorithmic information theory for the fundamental concepts in creativity and aesthetics.

This work is an extended version of our conference paper [13]. However, we give here a more complete argument and discussions on the following topics that we did not cover before: the *algorithmic complexity* and *probability* of an artifact’s existence, the various formulations of *model* and their role in defining non-randomness, *logical depth* as value and its non-equality to *compressibility*, *sophistication* as and encapsulation of core creator properties and its relation to *logical depth*.

## 2. Descriptional Complexity and Existence of an Artifact

### 2.1. Kolmogorov Complexity

We use this section to discuss the foundational Kolmogorov or Descriptional complexity. At the outset, we fix our computational model to be a Turing machine. The influential Church–Turing thesis [14,15] theorizes that any effective computation carried out by a human being can be simulated by a Turing machine. Although equating the creative process to the running of a Turing machine may seem reductive, we employ this computational model to enforce rigor and consistency. Moreover, it is not difficult to imagine a Turing machine playing the role of an inanimate creator that outputs a binary description of the creative artifact (e.g., pixel values of an image and a sheet music description of a composition).

The Turing machines that we work with are deterministic. This determinism helps one to pinpoint a machine that can absolutely generate an object *x* on some input in a finite amount of time with no uncertainty, which is essential for effective complexity analysis. These machines compute partial recursive functions and are defined only for some inputs, namely those on which the machines reach an accepting state. On the other inputs, the Turing machines either halt in a non-accepting state or run forever, and the function is undefined.

A Turing machine can be fully described by how many input, work and output tapes it has; the contents of the tapes; the positions of the pointers that move on the tapes; and the transition function. This description along with a corresponding input on which the Turing machine halts and outputs a binary version of an artifact *x* constitutes a concise functional description; we call this encoding a “program”.

These encodings or program descriptions of Turing machines are recursively enumerable: there exists a total recursive function that incrementally outputs all possible Turing machine descriptions (for a more detailed discussion on effective enumeration of partial recursive functions see section 1.7.1 of Li and Vitányi [7]) effectively enumerating all partial recursive functions.

An artifact in question can be an output of several of these Turing machines on appropriate inputs. It is important to distinguish between a function and the Turing machine or algorithm that computes it. For example, we can have a function that, on lexicographically increasing input *i*, outputs the $i^{th}$ letter in “War and Peace”, but there can be countably many number of ways in which this function can be computed. The shortest description for the object constitutes its absolute information content or Kolmogorov complexity [7].

To formalize this, we fix a Universal Turing Machine (UTM) *U*, which, given these programs, can simulate the corresponding machine on the encoded input. It is not difficult but tedious to imagine a UTM that accepts programs of form $1^{k}0i$ and acts as follows: it starts enumerating successive strings in ${\left\{0,1\right\}}^{*}$ and checks whether it encodes a valid Turing machine; if it does, then the UTM replaces a 1 in $1^{k}$ with a blank and continues.

Finally after finding the *k*th valid Turing machine, *T*, the UTM simulates it with the input *i* and behaves as *T* would on a representation of *T*’s tape contents. The powerful *invariance theorem* [7] states that there can be many such machines *U*, but that we should select one that is additively optimal—that is, the programs that it accepts are not too inefficient.

For example, a UTM may only accept programs that are padded with a constant number, *c* of zeroes, but they do not have any functional value. The Kolmogorov complexity of an artifact *x* is then the length of the shortest program that, when run on such a UTM, halts in finite time with output *x*.

(1) $$K\left(x\right)=\min_{p}\left\{|p|:U\left(p\right)=x\right\}$$

The function K(·) is not computable; there is not a single function *K*, such that it outputs the shortest program for all strings. A simple paradigm, like “enumerate and run all programs with lexicographically increasing length and the first program that halts with output *x* is its complexity”, does not work precisely because it is not decidable when or which program will halt. However, such absoluteness provides a lower-bound and a robust test-bed to evaluate and compare various information extraction methods.

To elaborate, the class of all description methods forms a hierarchy: some description methods take fewer bits to describe some objects than others. The foundation of AIT relies on certain niceness properties of this hierarchy; specifically it should have an unique minimal element that minorizes all other description methods. This unique element is that with which we define Kolmogorov complexity.

However, this does not stop us from having a fixed description method (artificial neural networks, context-free grammars, Huffman coding etc.) and comparing descriptions within that class [9,16]. For example: auto-encoders, when trained on a collection of paintings, attempt to concisely encode the data while minimizing the role of “noise” in it. The “size” of an optimal auto-encoder network can be used to approximate the dataset’s Kolmogorov complexity.

The usefulness of this description method depends on there being a certain computable minimal auto-encoding network that concisely describes the data, which gives us a lower-bound for comparison within this class. To compare this description method with another, say, a general purpose image compressor like JPEG, we package up everything a UTM (a typical daily-use computer) needs to decode the two description method.

For the auto-encoder, it might need the necessary Python and TensorFlow packages, and, for the JPEG, it would require the JPEG software to properly decode a compressed image. This way, we can always choose a suitable method to describe a particular data set that does not contain too much irrelevant information.

### 2.2. Algorithmic Probability

All of this background raises the question: Why should a concise description be preferred to one that contains redundancy? It is because concise descriptions are algorithmically more probable to be the artifact’s actual source. Here, we elaborate this.

If we write on the UTM’s input tape the results of an infinite coin-toss, and the UTM halts with output *x* after reading the first |p| bits, then the probability of *x*’s existence can be directly linked to probability that program *p* is a prefix of our infinite coin toss, $\frac{1}{2^{\left|p\right|}}$. The sum over all such programs $P\left(x\right)=\sum_{p}\frac{1}{2^{\left|p\right|}}$ can be regarded as the algorithmic probability of producing *x*.

However, consider the scenario where, for every artifact *x*, there is a Turing machine *T* that specializes in *x*, that is it outputs *x* for all inputs *i*. A program *p* that encodes this Turing machine and the inputs, has length at least |T|+|i|. Then, consider the total probability that our infinite coin toss generates this program:

$$2^{-|T|}\sum_{i\in {\left\{0,1\right\}}^{*}}2^{-|i|}=\infty \leq P\left(x\right),$$

which is unfortunate: P(x) is not a proper probability distribution.

To answer interesting questions like “What is the probability that *x* will be generated by the UTM?”, we need to go even further and require $\sum_{x}P\left(x\right)\leq 1$. This is solved by the Kraft inequality, which gives necessary conditions for an encoding scheme like this (e.g., each program is mapped to an artifact) to become a proper probability mass distribution [17]. It states that $\sum_{p}2^{-l(p)}\leq 1$ if the corresponding set of strings {p} is prefix free or uniquely decodable.

This can be achieved by padding the original *p* with ⌈log|p|⌉ bits of information about its own length: $p\to 1^{\left|p\right|}0p$, which makes the prefix free program much less likely to be generated by a fair coin toss and reduces its contribution to the overall probability. However, now the program encodes all necessary information needed to instruct the UTM on when to stop reading from its input tape and start executing.

Thus, we enforce the UTM to only accept prefix free encoding of programs and call P(x) the universal a priori probability, $Q_{U}\left(x\right)=\sum_{p:U(p)=x}2^{-|p|}\leq 1$. Equality holds if, for each valid initial segment, the UTM *U* halts with output *x*.

Being the shortest, the program *p* that witnesses the K(x) contributes $\frac{1}{2^{-|p|}}$ to $Q_{U}\left(x\right)$, which is larger than any other program, and thus *p* is the most probable source of *x* on the UTM. This rule implies that artifacts with short descriptions are overwhelmingly more likely to be produced than the ones with only longer descriptions, but one must also consider the fact that there are many more long programs than short one.

Consider string x:|x|=n with K(x)=logn+O(1) as highly compressible and strings with $K\left(x\right)=n^{1-\epsilon }$ as almost incompressible strings. Since there are overwhelmingly more incompressible strings than compressible ones and that counter-actively algorithmic probability favors those strings that have short descriptions, Woo [18] showed that following two results hold in the limit n→∞:

$$\left(i\right)\;E\left(\frac{\log n}{-\log Q_{U}}\right)=\infty$$

$$\left(ii\right)\;E\left(\frac{-\log Q_{U}}{n^{1-\epsilon }}\right)=\infty \mathrm{for}\mathrm{any}\epsilon >0,$$

where E(·) denotes the expected value. Then, (i) shows there is a non-negligible probability for the occurrence of highly compressible strings, whereas (ii) shows that there is also a non-negligible probability for the occurrence of nearly incompressible strings.

This prevents us from naïvely expecting that because there are more random strings the chance of finding a compressible string is exponentially small. Instead, the algorithmic a priori probability $Q_{U}\left(x\right)$ ensures that there are also factors working in favor of the occurrence of outputs with short programs. This notion of algorithmic probability becomes important again later when we talk about *logical depth*, the most probable computational explanation and the associated effort to generate an artifact.

## 3. The Order and Complexity of an Artifact

A useful derivative of Kolmogorov complexity is its conditional version K(x|y), which denotes the shortest program to generate *x* on *U*, which has been furnished with auxiliary information *y*.

(2) K(x|y)=min{|p|:U(p,y)=x}

Such a notion generalizes the unconditional definition as K(x)=K(x|ϵ) and also illustrates a natural way of describing objects with a predisposition to prior knowledge or inductive bias *y*. Additionally, the relation between K(x) and K(x|y) highlights *x*’s structure: if K(x|y)<K(x), then *x* and *y* share commonalities, while K(x|y)=K(x) means they are maximally different [7].

It is an elegant fact that using the conditional Kolmogorov complexity, we can express *x* with a two-part code, the first of which describes the regularities or recognizable properties *y* and the second part of which is the information exclusive to *x* [19]. This paves the way to robustly define the *typicality* and *novelty* of an artifact *x* with respect to *y* that concisely *models* a genre, a creator’s oeuvre, or a set of artifacts with which we want to analyze an unknown artifact with. The next three sections elaborate this idea.

### 3.1. Two-Part Code and Models

The plain Kolmogorov complexity K(x) lets us quantify the minimum information needed to create an artifact *x* on a UTM from scratch; the shortest program that witnesses this contains no redundancy; every bit of it is information needed for *x*. However, is it meaningful information? If *x* starts as a blank canvas and we flip a fair coin to determine each of its pixel values, then, with overwhelming probability, this painting constitutes its own short program; also with overwhelming probability, the painting is meaningless.

On the other hand, let *x* be a painting of rain where we determine the position of the rain drops randomly. Then, *x* can be described by the binary program pd where *p* is the non-random specification that *x* is a painting of rain and *d* is the accidental information containing the positions of the raindrops. This is formalized with a two-part code description of *x*: the length K(M) of the first part describing an appropriate *modelM* computed by a Turing machine, *T* and the length of the second part K(x|M) describing the left-out irregularities or random aspects of *x* after *Msqueezes out* the regularities of *x* [20].

(3) $$K\left(x\right)=\min\left\{K\left(M\right)+K\left(x|M\right):M\in \left\{M_{1},M_{2},\ldots \ldots \right\}\right\}+O\left(1\right)$$

Here, we use *model* as a hypernym to quantify the regularities in *sets of objects* and with which we recognize the regularities in *x*. The best model, *M* encapsulates the useful or compressible information in *x*, while minimizing the total description length. In relevant research [20,21], analysis has been mostly done with *M* denoting a finite set of objects. However, a model can also be a total recursive [22] or probability density function (PDF) [21]—each with its own properties and relevance. We discuss here the finite set and total recursive function frameworks, leaving the PDF approach for future work.

#### 3.1.1. Finite Set Model

The most natural way to formulate *M* is by a finite set of objects $\left\{x_{1},\ldots ,x_{n}\right\}$ signifying a history of observed phenomena with which a new object *x* is described. Following Ritchie [5], we call this the “inspiring set”. Then, K(M) is the length of the shortest program that generates the set and halts. The conditional information K(x|M) can be thought of *x*’s index in this set, as, with the set at our disposal, we only need d=log|M| bits to locate *x* in *M* (|d|=∞ if x∉M) completing the reconstruction. However, model *M* has to be such that the two-part code or cumulative information K(M)+log|M| through which *M* describes *x*, is no greater than K(x)+O(1).

Since *M* describes a set of objects, in most cases, it is almost bound to be larger than *x* itself (the complexity K(M) of a sensible model for a set of haikus will almost surely be larger than any individual K(x), as haikus are small objects and not very compressible [23]). Hence, a complexity restriction α is imposed on *M* such that K(M)≤α. This, however restrictive, ensures the model does not contain irrelevant information and also points to a lossy compression scheme of the set where it may no longer describe the set completely but only as allowed by α.

For example: *M* may describe “rain paintings”, and K(x|M), a specific one depending on the positions of the raindrops. If we are only allowed to use |p|=α bits to describe the painting to an observer, we may just indicate that it is a painting of rain and the particular positions of the raindrops may be chosen by the observer at random.

#### 3.1.2. Total Recursive Function Model

A more general class of models is the class of total recursive function *M* with which the issue of meaningful information versus accidental information is put in its starkest form. Here, the model *M* represents the projectable or shareable properties in a group of objects and its total recursive structure allows it to inject the properties into the input it receives to create more objects than the set it was built from. Thus, K(M) is the length of the most concise Turing machine that computes our inspiring set $\left\{x_{1},\ldots ,x_{n}\right\}$ such that there exist parameterizations $d_{i}:M\left(d_{i}\right)=x_{i}$ for all $x_{i}$.

**Figure 2:**
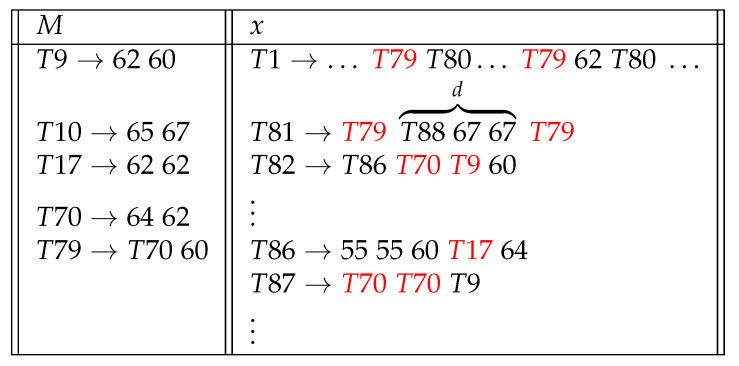
The left column is dictionary *M* of MIDI pitch patterns collected from a corpus of Beatles songs; the patterns exceed a certain occurrence threshold—they are frequently repeated in the artist’s songs. The right column is a song “Let it Be” from the same artist, with highlighted pitch patterns from *M* and the individual patterns that can be regarded as extra information *d*.

The input *d* can be found by letting *M* run on strings with lexicographically increasing length, and *d* is the first such string on which *M* halts and computes *x*; if no such string is found, then d=∞. Mondol and Brown [9] realized this modeling in the form of context-free patterns for a corpus of symbolic music: compositions from the same creator contain projectable pitch patterns that can be accumulated, and an unknown music string can be analyzed on the basis of whether or not it contains the patterns (Figure 2).

### 3.2. Randomness Deficiency and Typicality

We can associate a model *M*, however it may be defined, that describes a set of observed phenomena $\left\{x_{1},\ldots ,x_{n}\right\}$ with an observer’s familiar domain: the artists’ oeuvre, genres, collection of artifacts that share the same cultural contexts, etc. We can then measure how “typical” a new artifact *x* may seem to them with the “fitness” of the model *M* for *x*. Ritchie [5] used “typicality” to measure the extent to which a produced item is an example of an artifact class. We refine this argument with the *randomness deficiency* [20].

Let $M=\{M_{1},M_{2},\ldots \}$ be the set of finite sets containing *x*, where, for each $M_{i}\in M$, $K\left(x\right)\leq K\left(M_{i}\right)+\left|d\right|+O\left(1\right)$, where *d* is the extra information, $M_{i}$ requires to complete *x*’s two-part description. These $M_{i}$ are called algorithmic sufficient statistics [21]. These models allow a description of *x* with only a small increase in complexity while separating its randomness from the non-random part. The task still remains to choose the one among the candidates $\left\{M_{i}\right\}$ that *best-fitsx*, and, for that, we look at the second element of the two-part code, *d*.

Recall that, in the event where *M* denotes a finite set, it takes about log|M| bits to locate any x∈M (when *M* is a total recursive function, the extra information is the first input on which *M* halts with output *x*). This amount is called the data-to-model code length and is different from K(x|M). It is worthwhile to note here that, even though the two-part code is defined with the conditional complexity with respect to the model K(x|M) (Equation (3)), we make here the distinction between the raw input *d* to *M* and K(x|M), the latter of which is an input program fit for a UTM. If *M* instead consumed q:|q|=K(x|M), which is the shortest program for *x* given *M*, it would imply *M* is capable of running *q* and is a Universal Turing Machine itself, which it is not. On a similar note, if a model class does contain a universal model that can simulate all other models, this model class is not suitable for defining two-part codes consisting of meaningful and accidental information [19].

Since K(x|M) is the length of the smallest program outputting *x* given *M*, it leverages *M* and *x* in a way that could be much smaller than |d|. The difference between these quantities is defined as *randomness deficiency*, δ(x|M):

δ(x|M)=|d|−K(x|M)

**Figure 3:**
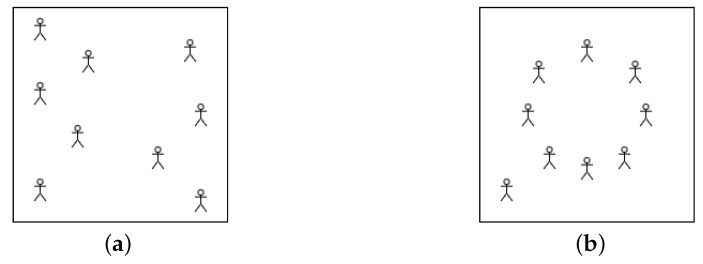
The children playing “Duck, Duck, Goose” are much more regularly positioned (**b**) than the randomly playing children (**a**).

A high δ(x|M) means that *x* contains more redundancy than captured by *M*; hence, *M* is not a best-fitting model for *x* and *x* is not a *typical* outcome of *M*. In other words, typical artifacts are maximally random with respect to the model that describes them, and we cannot significantly improve the conditional description of *x* given *M* than specifying its index or the input to it. Contemplate a model *M* that generates paintings of children playing on a playground after receiving their positions as input (Figure 3a). If we generate the input, *d* randomly, then, with overwhelming probability, K(x|M)≈|d|.

In this case, the painting has low randomness deficiency and is maximally typical relative to this model. However, if the painting, *x* we have is of children playing “Duck, Duck, Goose” where the player positions are less random (Figure 3b), the model no longer will concisely describe the painting. This is because, K(x|M) will make use of the information that a specific game is being played to compress *x* even further than it was possible for the previous painting. Moreover, randomly generated player-positions are much less likely to contain any regularities, which also makes *x* to be an unlikely output of *M*. We thus define the “typicality” of an artifact as follows.

**Definition** **1.** *Let M represent a minimal sufficient finite set model of $\left\{x_{1},x_{2},\ldots ,x_{m}\right\}$ such that $K\left(M\right)+\log|M|\leq K\left(x_{i}\right)+O\left(1\right)$ for 1≤i≤m. Then, the typicality of an object x with respect to this model is as follows.*

(4) typicality (finiteset) (x|M)=−δ(x|M)=K(x|M)−log|M|

*Let M represent a minimal sufficient total recursive function model of the inspiring set $\left\{x_{1},x_{2},\ldots ,x_{m}\right\}$ such that there exist parameters $\left\{d_{i}\right\}:M\left(d_{i}\right)=x_{i}$ and $K\left(M\right)+|d_{i}|\leq K\left(x_{i}\right)+O\left(1\right)$ for 1≤i≤m. Then, the typicality of an object x with respect to this model is as follows.*

(5) typicality (totalrecursivefunction) (x|M)=−δ(x|M)=K(x|M)−|d|

When *x* is not in *M*’s range, then |d|=∞ and we find the lowest typicality −∞. If typicality is close to the maximum value 0, then there are no simple special properties that single *x* out from the majority of elements in *M*. Note that, based on *M*, the typicality that *x* exhibits can be akin to either *H*-creativity (producing an idea/artifact, which is wholly novel within the culture, not just the creator’s oeuvre) or *P*-creativity (producing an idea/artifact, which is original as far as the creator is concerned) [24]. However, the analysis we present here is effective for understanding both, if *M* contains members that are consistent with the class that we are interested in.

### 3.3. Novelty

The notion of “novelty” is related to but not exactly “atypicality”, the inverse of “typicality” as we discussed above. We need additional properties of the object being a surprising or unexpected outcome of the contemplated model. McGregor [25] addressed this idea by defining the novelty of an object by looking at its information distance [26] from each of a collection of objects of the same class and choosing the minimum of these distances as the novelty of the new object. In the same paper, the author critiqued the idea by pointing out that the observer estimating novelty needs to have a perceptual frame in which to work. This approach can be fleshed out using the model and data-to-model code that we described earlier.

The central motivation in our two-part code discussion has been to find the true source for *x*, which we try to achieve by modeling the data at hand as best as we can. However, there is a non-zero probability that the outcome of 100 fair coin-flips is 00…0. A modeling method that identifies flipping a fair coin as the cause of this outcome is surely a bad method, even though the source of the data it came up with happens to be the true cause. However, in real-world problems, such as modeling creative products, the data can be just unique or accidental for the model that actually produced it.

A monkey hitting keys at random on a typewriter keyboard for an infinite amount of time will almost surely type any given text, such as, the complete works of William Shakespeare. An observer after examining the first few thousand outputs of this typing will create a model, “monkey typing on typewriter”, but if the next output is the first page of Hamlet then it will surely come as a shock to them.

In this case, the accidental element is in the raw data, *d* that the monkey-model is supposedly printing out and the fact that the current data is so different from the previously observed data. Thus, for novel and unexpected artifacts the model might still describe the regularities in the object; but, the extraordinary conditions or the data-to-model code that caused the model to output it, sets the object apart from other objects created by the same model.

Let model *M* be a minimum sufficient statistic for the inspiring set $\left\{x_{1},\ldots ,x_{m}\right\}$. Then, there exist parameters $d_{i},d:M\left(d_{i}\right)=x_{i},\;K\left(M\right)+\left|d_{i}\right|\leq K\left(x_{i}\right)+O\left(1\right)\mathrm{for}1\leq i\leq m$. An artifact *x* is novel with respect to a model, *M*, if it is an unlikely and original outcome of the model. The object, *x* may be still producible from model *M* (just like “Hamlet” is producible from a “monkey hitting keys on a typewriter”, but with astoundingly low probability), but the remaining distinctiveness d:M(d)=x is an indicator that there are better models to produce *x* (consider the model *M* accidentally finding a set of ordered locations to place the children in the playground when location are being randomly generated).

Model *M*’s unfitness is captured by *x*’s reduced kinship with other artifacts $\left\{x_{i}\right\}$, using which *M* was created. Note that, *M* is the common information between the novel object and the inspiring set, and the data that set them apart are the inputs $\left\{d_{i}\right\}$ and *d* to the model *M*. Thus, the extent to which *x* is novel is determined by how much information in shared between $\left\{d_{i}\right\}$ and *d*.

The novelty of *x* is then essentially captured by the mutual information $I\left(\left\{d_{i}\right\}:d\right)=K\left(d\right)-K\left(d|\left\{d_{i}\right\}\right)$ between *d* and $\left\{d_{i}\right\}$ (Li and Vitányi [7], p. 249). The less $\left\{d_{i}\right\}$ informs on *d*, the more novel *x* is, reaching its maximum at $I(\left\{d_{i}\right\}:d)=0$. On the other hand, if $d_{i}$ and *d* are similar in structure, it indicates that the individuality that *x* possesses after *M* has extracted its regularities, is not unique. Hence, the object is not surprising, which could implicitly mean that the randomness deficiency δ(x|M) is large. However, $I(\left\{d_{i}\right\}:d)$ measures the difficulty with which we figure this unfitness out even with the available information.

**Definition** **2.** *Let M represent a minimal sufficient total recursive function model of the inspiring set $\left\{x_{1},x_{2},\ldots ,x_{m}\right\}$ such that there exist parameters $\left\{d_{i}\right\}:M\left(d_{i}\right)=x_{i}$ and $K\left(M\right)+|d_{i}|\leq K\left(x_{i}\right)+O\left(1\right)$ for 1≤i≤m. Then, the novelty of an artifact x, which may or may not be in the inspiring set but is producible from M with parameter d:M(d)=x, is*

(6) $$\mathrm{novelty}\;\left(x|M\right)=-I\left(\left\{d_{i}\right\}:d\right)=K\left(d|\left\{d_{i}\right\}\right)-K\left(d\right)$$

Note that, our *mutual information* formulation of novelty is related to the *information distance* formulation by McGregor [25], as the information distance increases when mutual information decreases in general. However, our approach can model a “perceptual frame” to analyze an artifact, which cannot be done using the information distance between pair of artifacts. This was a limiting concern for McGregor himself [25].

Novelty can thus be compared to explorations undertaken by an artist that remodel our understanding of a genre or a pre-conceived idea of a category. Learning and applying new techniques as well as exposure to new environments and influences, can bring about novel objects that capture the general spirit of the inspiring set but are difficult to be recognized in their light. John Cage’s 4’33” [27] is a prime example of such an artifact; it is one of the most influential yet controversial classical music pieces of the 20th century [28].

## 4. Value as Computational Effort

In addition to the length, a program has other important properties that let us evaluate the overall creation process of an artifact. For example, when the UTM runs the program, how much of its tapes are occupied at any given time, whether the steps it takes are random or logical and how many logical steps at minimum is required to output an artifact *x*. Answers to these questions let us measure the minimum amount of resources needed to generate an artifact and this is non-trivially connected to the “value”, “quality” or “usefulness” assignable to the creative artifact. This section presents this argument.

### 4.1. Logical Depth and Its Relation to Value

The length of a program that generates an artifact is not a sufficient indication of the computational complexity that is associated with the generation process. Complex computations can be expressed concisely—more so if the program that is undertaking the mammoth task is also the one that witnesses the Kolmogorov complexity of the artifact or close to it in size. An object like DNA, is something really simple but disguised by complicated manipulations of nature or computations by a computer.

This computational “effort” to generate an artifact from scratch can measured be in time, space or energy required by the program. All of these are resources that we may have restricted access to. As such, we tend to naturally value an object for which an extraordinary amount of these resources was needed to create it. However, a computer that embarks on a long computation will naturally radiate heat when it manipulates or erases information [29]; to cool it down, we need to spend energy still, making the spent energy almost proportional to the spent time.

The discussion on assigning value to space is rather tricky; as space can be reused without extra cost and even the polynomial space complexity class PSPACE contains some of the most difficult problems (P⊆NP⊆PSPACE [30]). As such, we suggest that it is more interesting to discuss problems with bounded time.

Note that, due to the incomputability of K(x), we cannot know the actual time required by the shortest program for it. Rather, we decide for each program when to stop its running by defining a computable time bound t(|x|) as a function of the artifact’s length, as it is natural to assume that a program will take less time to print a 8-bit-long string than to print a 512-bit-long one. The important key here is that we know when to halt the program using the computable property of the time bound.

We then define here a time-bounded version $K^{t}\left(x\right)$, which is the length of the shortest program *p* that outputs *x* within time t(|x|).

(7) $$K^{t}\left(x\right)=\min_{p}\left\{|p|:C\left(p\right)\mathrm{halts}\mathrm{with}x\mathrm{in}\mathrm{time}\mathrm{at}\mathrm{most}t(|x|)\right\}$$

Intuitively, as t(|x|) becomes larger, the length of the program that needs this time can become shorter, as it takes more time for them to create a large artifact than it takes an already larger program. In other words, $K^{t}\left(x\right)$ suffers from $K^{t}\left(x\right)-K\left(x\right)$ *redundancy* or *ad hocness*, as $K^{t}\left(x\right)$ may have to store some information about *x* without meaningfully compressing it and decompression from a shorter description may require time that we do not have. This difference is known as the Computational Depth of *x* [31].

(8) $$cdepth_{t}\left(x\right)=K^{t}\left(x\right)-K\left(x\right)$$

As *t* grows, excluding the pathological cases (programs doing unnecessary computations), the non-randomness in *x* becomes disguised by complicated manipulations or computations by the program.

However, it is not always economical to sacrifice in time for a small gain in length. In Conway’s game of life, a simple initial configuration can give rise to non-trivial organized complexity after running for an exponential amount of time. This may lead us to think that this final configuration is valuable: it is attainable from its initial simplicity but only with great endeavours.

However, if it can be reached from other slightly more complex initial settings within polynomial time, then it no longer seems as valuable as it did before. Therefore, a more appropriate way of defining value is: an object is valuable only if *all* of its short programs are slow-running. In other words, almost all of its *algorithmic probability* comes from slow and short programs. This is captured succinctly by the *logical depth* [32], which we adopt as a formal measure of *x*’s aesthetic value.

**Definition** **3.** *The value or quality of a creative product x is the minimum computational effort or time needed to produce it from an s-significant short description.*

(9) $$value_{s}\left(x\right)=depth_{s}\left(x\right)=\min_{p}\left\{time\left(p\right):U\left(p\right)=x\mathrm{and}|p|\leq K\left(x\right)+s\right\}$$

Here, *s* denotes the significance of the witness program, *p* in terms of its contribution $\frac{1}{2^{K(x)+s}}$ to *x*’s existence or *algorithmic probability*: the less *s* is, the more probable it is for *p* to be *x*’s actual source on *U*. Thus, *p*’s computational effort is also increasingly close to the most probable way in which *x* was created. Among these available candidates, the minimum time taken by them is considered to avoid selecting programs that despite producing the desired object do so inefficiently, whereas other similar-length programs are faster. High computational depth is an indicator of high logical depth; however, the latter is a stronger concept as it weighs programs by their contributed algorithmic probability $\frac{1}{2^{K(x)+s}}$ and conservatively selects the least time spent by these programs in order to avoid overestimating the artifact’s computational effort.

### 4.2. Compressibility ≠ Value

We propose that what makes a creative object valuable is not its information content, but rather the amount of computational or creative work it relieves its receiver from repeating, which was plausibly done by its originator. A sequence that represents the outcomes of *n* coin tosses, has high information content but little value.

Conversely, a book on algebra may list a number of difficult theorems, but has very low Kolmogorov complexity, since all the theorems are derivable from the initial few definitions and axioms. However, such derivations can be time-consuming and if we transmit only a short description containing the theorems of the book, a receiver has to spend a long time to reconstruct their proofs. Sending the entire book does not increase the information content transmitted, but now the receiver has all the useful information readily available for adaption.

Thus, the value of an object does not depend on its absolutely unpredictable parts (information content), nor on its obvious redundancy (verbatim repetitions, for example), but rather on what might be called its buried redundancy— parts reproducible only with difficulty, things the receiver could, in principle, have figured out on their own, but only at considerable cost in resources or computation [32].

Note that such definition of value also protects us from certain pitfalls; like claims that highly compressible and regular objects are valuable. Rather, we seem to attach value to objects that are meaningful, and often such meaning is acquired over time. In Colton’s Art exhibit example, a similar scenario is described where an art enthusiast deems a painting of random dots more valuable than another similar painting, as it had a meaning attached to it provided by the painter [33], which is that “the random dots depicted his personal relationships, and the color and positions of the dots symbolized how he felt about them”.

This allows a more succinct description of the painting than the verbatim description of the dots’ positions and color, as the profiles of the dots are no longer random. Moreover, such a painting would be impossible without the author building the relationships throughout their life.

**Figure 4:**
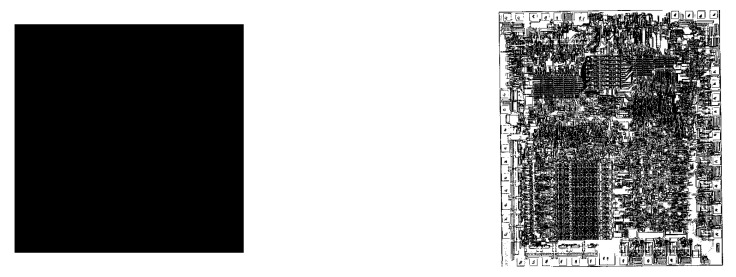
The mono-coloured image on the left is more compressible than the complex one on the right; yet, the latter takes more decompression time from its short program. Image taken from: Zenil *et al*. [12].

In an interesting study, Zenil et al. [12] showed that complex but non-random images that we would normally assign value to takes more time to generate from their lossless PNG (Portable network graphics) compressions (Figure 4).

**Figure 5:**
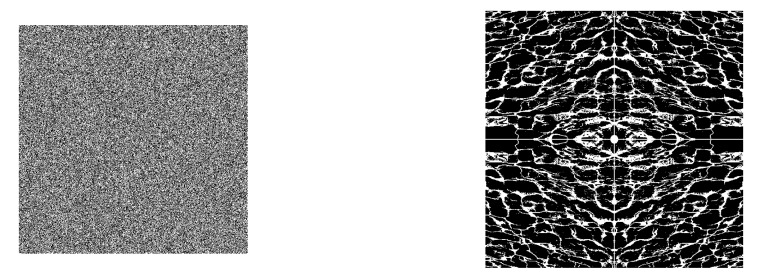
The image on the left is purely random noise. Hence, a suitable program that generates it will contain a lot of verbatim descriptions, taking little time to reproduce the image. The image on the right, by the virtue of being highly compressible, takes more decompression time from its short program. Image taken from: Zenil *et al*. [12].

On the other end of the spectrum, highly random objects, which are not compressible and are often of little value to us, can be generated quickly from their similar-length programs (Figure 5).

It is important to note here that this object-dependent notion of value does not consider the cultural context in which it was conceived nor the observer’s perspective, which may differ from person to person. Conceivably, much of this cultural background knowledge could also be modeled [34]. Instead, what it correctly predicts is that valuable objects are extremely rare: there are far more random objects in the universe than there are highly regular compressible ones; even less are among those which are programs and go through a long derivation to create something meaningful.

This takes us to the discussion of creative process and the importance of the logical steps taken in an artifact’s generation.

### 4.3. The Logical Steps of a Creative Process

We highlight here one more aspect of logical depth—its ability to identify the most likely and effective generation process for an artifact in the presence of multiple plausible theories. A logically deep object is an outcome of a long, non-random and non-trivial computational process, and the evidence of this becomes expressed through the artifact’s subjective organization. Any ad hoc or random step involved in the process will necessarily increase the program length that witnesses such process, making it a less viable source for the artifact.

In “The Library of Babel”, a short fiction by Borges [35], the librarians attempt to discern which of the books are meaningful in a library that contains all possible 410-page books with a 25-letter alphabet. This exhaustive search that leads to finding a meaningful book, is indicative of the object’s non-random generation process. The short program that encodes this search is not much larger than the meaningful short program that actually produced the book and so can be counted among its plausible sources.

Moreover, this is similar to the *diagonalization* process of creating logically deep or valuable artifacts [32]: consider generating an artifact *x* with value *T* (some large number). We enumerate all programs p:|p|≤T, run each of them for time *T*, check whether the cumulative probability $\sum_{p:U(p)=x}2^{-|p|}$ of programs that halt with output *x* is significant or larger than $2^{-|x|}$ (which means *x* is compressible and has a more probable source than a verbatim description).

If we do find an object *x* that is less probable to be created by these *T*-fast programs, then we can say with high probability that *x* has value at least *T* (as programs taking more time than *T* may be able to produce *x*; the algorithm we just described is certainly one among them). This is a very slow process, taking more than $2^{T}$ steps to create an object with value *T* (similar to going through a large number of books in “The Library of Babel” to find a meaningful one).

However, this process cannot be sped up due to the *slow-growth law*, which states that a fast deterministic process cannot turn a shallow or valueless object into a deep and valuable one [32]. If it did, then this process along with the shallow object (can be an empty string) could be encoded as a program that generates the valuable object quickly, contradicting the fact that the object is valuable.

A delightful example of non-trivial creative process is yet another short story by Borges [35], “Pierre Menard, Author of the Quixote”, where the author writes about Menard’s great effort to recreate, line for line, the first few chapters of “Don Quixote”. Menard does this solely from an intimate knowledge about the book’s context and meaning—which Borges implies as being much shorter and more concise than the book itself.

On the other hand, logically shallow artifacts can be produced with little effort by programs that are not too much larger than the shortest one. A fraudster or charlatan may claim that a complex-looking creative product is a result of a slow-running short program, thus, artificially inflating its value. However, if the object has an equal-length fast program, which involves no ad hoc steps in generating the object, then it is equally likely that the latter is the true generative process for the object. If the object *can* be produced by taking a small number of non-random steps, then it is certainly possible that the fraudster program takes unnecessary pathological steps in order to seem serious.

In Colton’s Dots example, a dishonest painter can masquerade a truly random painting as a valuable one by supplying the observer with a short program that *pretends to* simulate the events and relationships of the painter’s life up to the point of creating the painting, which could require exponential time to halt [33]. However, an observer with limited time but access to the logical depth of the painting (through an orcle) can quickly disregard the false claim by generating a fast program that outputs the painting with a length close to the given pretend program.

Hence, the most plausible creative process is carried out by a program that is *s*-close to the shortest one for a fixed and small *s*. In addition, the work to reproduce *x* from the program cannot involve any unnecessary, ad hoc assumptions except for the *s* bit redundancy; if it does, then it will no longer be *s*-significant.

**Definition** **4.** *An s-significant creative process of a product x is simulated by the UTM upon input p such that |p|≤K(x)+s, U(p)=x and p takes the minimal non-random steps among all s-incompressible programs for x.*

If a short program *p* has a slow deductive reasoning process, it is not evidence against the plausibility of this program. In fact, if the product has no comparably concise programs to compute it quickly, this is evidence of the non-triviality of the generative process. A great work of autobiography is one example of this: if we just consider the written text as its acceptable representation, then its information content is low [36]. However, the existence of such literature stands evidence of a profoundly-led life by the author and the significance of the events that happened within that lifetime.

## 5. Sophistication and the Creator

We now discuss another exciting idea in AIT, *sophistication*, which lets us formalize the characteristic styles and creative properties that a creator leaves in the artifacts they generate. This is analogous to *effective complexity* defined by Gell-Mann and Lloyd [37] in an effort to separate regularities from randomness. In their work, they advocate for considering a work to be of quality if its regular parts are complex:

“We may call a novel complex if it has a great many different characters, scenes, subplots, and so on, so that the regularities of the novel require a long description.”

This section formalizes this notion along with a mechanism for identifying *masterpieces*, artifacts that have a maximal amount of non-random regularities.

### 5.1. Generative Attributes of a Creator

In Section 3.1.2, we talked about formulating a model with a total recursive function, which generates a set of inspiring objects on appropriate inputs. Here, we reintroduce total recursive functions but furnish it with a different purpose—to capture the structural information in an individual object *x* that shows evidence of some planning that went into *x*’s generation.

In our discussion of *logical depth*, we have seen that the complexity and meaningful information in an object do not have a causal relationship; in fact evidence suggests that they may very well be orthogonal to each other [22]. The notion of *sophistication* is an attempt to decouple the part of an object that is an aggregate of *shareable* or *projectable* properties from its incidental information with a two-part code.

This is not too different from our discussion on total recursive model but here the focus is on extraction of such structure from the object itself and its relation to the source of the object. The total program *p* that witnesses the *sophistication* of an artifact, needs |d| extra bits to complete a two-part description (Equation (10)) of the artifact. The data-to-model code length |d| helps determine the *typicality* of the artifact with respect to the total program *p*.

In optimal cases, where the two-part code length |p|+|d| is not too large (a constant *c* away) from the artifact’s Kolmogorov complexity K(x), |p| offers an upper-bound on the structure present in the artifact [19]. In this case, the artifact has low randomness-deficiency or is *typical* with respect to this program [38]. If, instead, it had high randomness deficiency or larger |d|, the *c*-closeness to K(x) will have to be violated.

Originally, sophistication was also defined as a monotonic function: on larger inputs, the total function should be able to produce larger outputs with the same property [22]. This helped define structure in infinite objects for which the program length stays the same while the input becomes larger. However, more importantly, it allowed a way to assign *intelligence* to the artifact’s creator. Consider a music generator, which is broadcasting self-composed music and whose inner mechanism is unknown to the observer.

If the composition obeys some simple rule, such as repeating the same patterns or sounds maximally random, then we would not attribute intelligence to the source. If, however, the composition exhibits complex structure, which is only possible through rigorous planning and meaningful exploration, we might suspect the existence of an skilled creator. Hence, sophistication is that quality of an object that sets apart the artist’s talent from their incidental impulses.

**Definition** **5.** *The creative quality of the creator inherent in an artifact x is measured by the object’s c-significant sophistication $soph_{c}\left(x\right)$. That is, we can say that the generator program p of length $soph_{c}\left(x\right)$ produced x on input d leaving out all but c bits of redundancy.*

(10) $$\begin{array}{cc} \mathrm{Creator}\mathrm{Attributes}(x)= & soph_{c}\left(x\right)=\min\{|p|:p\mathrm{is}\mathrm{total},a\mathrm{parameterization}d\\ \; & \mathrm{exists}\mathrm{for}\mathrm{which}U(p,d)=x\mathrm{and}|p|+|d|\leq K(x)+c\} \end{array}$$

Here, the significance parameter *c* calibrates the redundancy the total description length |p|+|d| is allowed to have; it may be *c* bits longer than *x*’s shortest description. However, in order to reduce *c*, if we furnish *p* with properties that are accidental or exclusive to *x*, then it might fail to recognize objects that are generated by the same source. Hence, the significance parameter *c* is interpreted as a confirmation of the description (p,d) before regarding extra structure represented by a longer program $p^{\prime }$.

The utility of such formulation to define a creator becomes further evident through the following example: consider a total function backward(x) that on any input *x*, reverses the string ($\tilde{x}$) and outputs $x\tilde{x}$: backward(011)=011110, backward(0010)=00100100. Such properties are often difficult to find and will be overlooked by a mechanism that tries to find superficial patterns (e.g., LZ78), but work as an excellent compression scheme for the product for which they are defined.

However, more importantly, such functions have generative qualities. If we enforce properties, such as *injection* or *monotony* on the total function, we essentially create a method that can generate creative artifacts with a certain style embellishments (*p*) upon receiving influences (*d*) from the environment. Furthermore, by using a UTM with two prefix-free input tapes, we can analyze how likely an artifact *x* is a product of a creator simulated by a program *p* by analyzing the likelihood of obtaining a prefix-free *d* in fair coin tosses [23,39].

#### Computability

The idea of sophistication is, of course, not without challenges: Grover [40] reports that it is not possible to fully separate the structure from noise in an object and, thus, sophistication is not objectively quantifiable. Gell-Mann and Lloyd [37] reiterates this concern by noting that depending on the person or machine trying to capture the regularities, different properties of the artifact may present themselves. For example, a necktie designed by Jerry Garcia may be described by its complex patterns. However to a dry cleaner, the coffee and wine stains on the necktie may appear to be more prominent features, as to them the origin of the tie is its owner [37].

Rather, for a fixed UTM and *c*, sophistication provides a theoretical upper bound on the structural properties of a string [41]. Sophistication is non-increasing in *c* and for c=|x|+O(1), sophistication is bounded by log|x|. This is because with a large *c*, we can find a total program that tells a trivial “*print*” program to read the first ⌈log|x|⌉ bits of the data, which is now the raw *x* and output it. However, is it possible to arrive at a non-trivial total function? The answer lies in its relation to *logical depth*.

Both logical depth and sophistication are measures of meaningful complexity in an object. One uses dynamic resources (program time), the other uses static ones (program size). Thus, the two measures are not necessarily correlated [42], because a short unassuming program can take a long time. An example of this is the characteristic sequence of the diagonal halting problem, χ, where each bit χ[i] is 1 if the *i*th program halts. Despite its apparent importance, the *n*-bit prefix $\chi_{n}=\chi \left[0\ldots n-1\right]$ of χ is highly redundant with $K\left(\chi_{n}\right)=\log n+O\left(1\right)$.

The intuition is that we only need to specify the *number* of indices that contain 1. Once this logn number is known, we can dovetail all the programs $p_{0},\ldots ,p_{n-1}$ on an UTM and stop the computation once the desired number of programs have halted [7]. This procedure can be easily converted to a total recursive function that on input logn prints out the first *n* bits of the halting sequence, so its sophistication is low. Yet, this is computationally very expensive, taking at least as much time as the slowest program in the above enumeration requires to halt.

Rather, logical depth can be used as a mechanism to find structure. We formalize this with the converging hypotheses argument [22]: consider the same music generator as in the previous section. As we observe more of its music, more structure becomes apparent, thus, forcing revisions of hypotheses as to the generator’s structure. Let *x* denote the complete music and $x^{n}$ its observable prefix. At step *n*, we would like to find the hypothesized generator $p_{n}$ and its parameterization $d_{n}:p_{n}\left(d_{n}\right)=x^{n}$ and $|p_{n}\left|+\right|d_{n}|\leq K\left(x^{n}\right)+c$.

If for the previous hypothesis $p_{n-1}$, we find an input $d_{n-1}:p_{n-1}\left(d_{n-1}\right)=x^{n},|p_{n-1}\left|+\right|d_{n-1}|\leq K\left(x^{n}\right)+c$ then we move onto a larger prefix without updating the hypothesis. Note that, this input will be easy to find if the function is *strictly monotonic* [22]. Otherwise, we exhaustively search through all programs (not necessarily total) p:|p|≤n and data d:|d|≤n−|p| in order of increasing length and let them run for $ldepth_{c}\left(x^{n}\right)$ steps.

We choose the shortest $p=p_{n}:|p|+\left|d\right|\leq K\left(x^{n}\right)+c$ that satisfies these criteria. This way, as more composed music is revealed, previous hypothesized generators are abandoned for one of two reasons. The most straightforward reason is that the subsequent parts of the composition is inconsistent (do not fall in the generator’s range). In this case, the program is changed in favor of one which is less powerful (shorter, using longer data). The other reason for abandoning a program is that as more parts of the composition is observed, structure becomes apparent, which was not previously so; that is, use of a more powerful, longer program results in a shorter description when including the required input to generate $x^{n}$.

Such a procedure might not give the smallest compression program for the music generator itself. However, it increasingly describes the properties of initial segments of its generated music *x*, which can be used to compress the larger initial segments increasingly better as n→∞.

### 5.2. Non-Stochastic Objects or Masterpieces

Sophistication is also a natural way to measure how much information of an artifact we can throw away without losing the ability to query its properties (without false positives). These properties constitute the *non-stochastic* part of an artifact and a remarkable outcome of this notion is *absolutely non-stochastic* objects [21]. These are *highly sophisticated* artifacts whose non-stochastic properties contribute to almost all of their complexity. We now show that *creative masterpieces* fall into this category

An absolutely non-stochastic object, whose complexity is mostly comprised of non-random structure, has neither minimal nor maximal complexity. Hence they are not typical outcomes of any total recursive program that exhibits low structure (e.g., model that outputs random outcomes of fair coin tosses or strings of 1 s). Additionally, non-stochastic objects have no optimal programs that are of relatively small complexity, that is they exhibit high randomness deficiency or atypicality for any program *p* with K(p)≪K(x).

Rather, these objects are typical outputs only of programs *p* that have complexity close to their own, K(p)≥K(x)−O(1) [21]. Non-stochastic information is highly improbable to be generated through a random process. Thus, they cannot contribute to the data portion of the two-part code in a short program and must be included in the non-random model part.

Thus, the program *p*, which witnesses the sophistication of such an artifact showcases the creator’s elaborate techniques, mastery and creative properties that can be pioneering and transformative. When a non-stochastic object is an output of a unique and highly sophisticated program, it is ambitious in scale and depicts innovation, pushing a medium or genre to new directions. Hence, absolute non-stochasticity is a pre-cursor to creative masterpieces.

**Definition** **6.** *A creative masterpiece x is absolutely non-stochastic or highly sophisticated, that is, they exhibit low randomness deficiency (needing small additional data) only for total recursive programs p that have complexity close to their own, K(p)≥K(x)−O(1). For programs p with K(p)<K(x), they will either require large additional data or they will not be in those programs’ range at all.*

If we were to partition a creative masterpiece into meaningful complexity and random noise, we will find that almost all of its complexity comes from useful incompressible properties. Moreover, the amount of such non-randomness is also significant. Thus, non-stochastic artifacts reside in a Goldilocks complexity zone: Shen [43] showed that these objects have complexity of at least $K\left(x\right)\geq \frac{n}{2}-O\left(\log n\right)$. This lower-bound for complexity helps distinguish a sophisticated artifact from the rest of the creator’s oeuvre.

Let *S* be a finite set that is a minimal sufficient statistic for *x*, which means x∈S and K(S)+log|S|=K(x)+O(1). Consider the other artifacts $\left\{x^{\prime }\right\}$ in *S*, for which *S* is a sufficient statistic; that is, the artifacts are *typical* examples of *S*. If they have length much larger than |x|, but $K\left(x^{\prime }\right)\approx K\left(x\right)$, then we cannot say $x^{\prime }$ is a *masterpiece*, as for these objects, $K\left(x^{\prime }\right)\ll \frac{|x^{\prime }|}{2}-O(\log|x^{\prime }\left|\right)$. However, since these objects are compressible and exhibit substantial non-trivial structure *p*, they can be considered “good” artifacts. Thus, a *masterpiece*, which is a product of its generative program *p* having relatively high complexity while being absolutely non-random, is an extremely rare phenomenon.

#### High Sophistication = Strong Depth

A result by Antunes and Fortnow [42] shows that artifacts with high sophistication that is, $soph_{c}\left(x\right)>n-2\log n-2c$, are also *logically deep*. This can be shown by a *diagonalization* argument, which we briefly state here: since highly sophisticated artifacts have total recursive programs p:|p|>n−2logn−2c, we enumerate all the artifacts that can be produced with a total recursive program q:|q|≤n−2logn−2c and inputs d:|d|<n−|q|−c within some really large but finite time bound, *T*.

Let *T* be the maximum time taken among all the *q* and *d* pairs. If we enumerate all artifacts that have *q* and *d* as their two-part code, which halts within the time-bound *T*, we have created all the artifacts that are not sophisticated. Moreover, these artifacts are *T*-shallow. Hence, lexicographically the first artifact that is not in this set is both sophisticated and logically deep. This is also fortified by Joosten et al. [44]: the average runtime of Turing machines computing a function increases, with probability close to 1, as a function of the number of states.

This indicates that, machines not terminating (almost) immediately tend to occupy all the resources at hand. In other words, sophisticated objects, which require complex programs and cannot be generated by simpler ones, also require long computations by those programs.

This interplay between complexity of machines and difficulty of execution takes an extreme form in *strongly deep* or *transcendent* objects [22,32]. These objects exhibit unbounded quantity of inner structure even after an observer spends a considerable amount of time looking for such structure (i.e., basis for compression) [45]. More formally, let t(n) be any recursive and reasonable (does not grow too fast with *n*) time bound with |x|=n. Then, any observation $K^{t}\left(x\right)$ of a *strongly deep* product *x* will still exhibit high computational depth $K^{t}\left(x\right)-K\left(x\right)$ (Section 4.1, Equation (8)).

This is comparable to a synopsis of a great novel, which, in principle, would be insufficient to exhaust its value, as significant portion of the novel’s meaningfulness will likely remain undiscovered by it. Similarly, the extent of computational effort and resources needed to recreate a creative product nearly identical to the highly sophisticated artifact, alludes to its strong depth or quality.

The documentary “Tim’s Vermeer” [46] contains one such example where an American inventor, Tim Jenison with intimate knowledge about Vermeer and his techniques, attempted to reproduce “The Music Lesson” [47] over a span of almost six years. Regardless of the painting’s artistic proximity to the original, the question remains whether such a recreation is able to exhaust the original’s value [48]. Such effort is indicative of the resources at the minimum needed to represent a great artwork as such.

## 6. Related Works and Discussion

With all of the background of Section 2, Section 3, Section 4 and Section 5, we now present some prior research on computational aesthetics and discuss how some of formulations can be reviewed using the tools we discussed above.

To the best of our knowledge, the current work is the first detailed look at the relationship between some of fundamental ideas in computational creativity (typicality, novelty, value, creator-properties and qualities of masterpieces) and their counterparts in AIT. Significant earlier work can be attributed to Moles [49] who examined the role of classical information theory in aesthetic perception: an observer selects and abstracts structure from their sensory input (a picture, a musical sequence, a written text) and the “selective information” and “redundancy” in the received message are largely related to intuitive quantities like “degree of unexpectedness” and “originality”.

The author views meaningful messages as being invariably embedded in a background of “noise” and that an observer’s perception actively selects the meaningfulness from a muddled context. Prior to introducing information theory in this field, Birkhoff [50] examined the formalization of the aesthetic measure *M* of an object with the ratio of its “order” (*O*) and “complexity” (*M*), $M=\frac{O}{C}$.

The specific definition of *O* and *C* depends on the type of analyzed objectl however, it must ensure that the resulting *M* reflects the aesthetic quality of the object. This led Birkhoff to define complexity as the number of units in the object, which require a conscious act of attention (e.g., the number of tones in a melody or number of edges of a tile), whereas order is characterized by the pleasant feelings associated with the basic properties of objects, such as symmetry, repetition, similarity and balance.

Later, Rigau et al. [51] further formalized the ratio *M* with a normalized redundancy or compressibility ratio. This is achieved by defining complexity with an encoding length of the object: e.g., sRGB color representation of a picture and order with compressibility, which is measured as the difference between the above encoding and the optimal encoding using some compression scheme, e.g., PNG compression of images.

**Figure 6:**
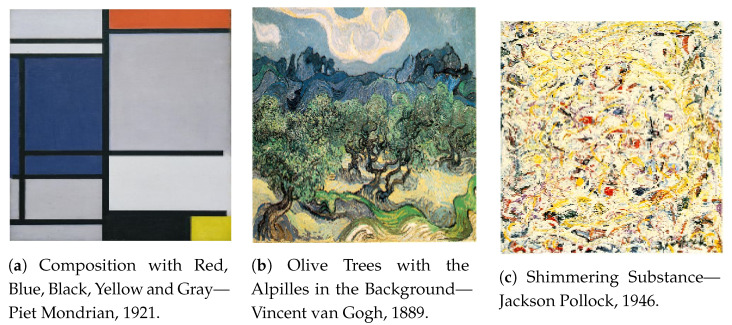
(**a**) contains considerably more order than (**b**,**c**). Image taken from: Rigau et al. [51].

They show experimental results that such measure assigns a higher *M* to Mondrian’s painting than it does to Van Gogh’s and Pollock’s paintings, which is due to the latter artists’ works’ relative lower compressibility (Figure 6). Several other authors have introduced different measures with the purpose of quantifying aesthetics: Kosheleva et al. [52] consider the running time t(p) of a program *p*, which generates a given artifact as a formalization of Birkhoff’s complexity *C*, and a monotonically decreasing function of the length of the program l(p) (i.e., an approximation of Kolmogorov complexity) represents order *O*.

Thus, the aesthetic measure is defined by $M=\frac{2^{-l(p)}}{t(p)}$, and a measure of “beauty” of the object is the smallest value of the product $t\left(p\right)2^{l(p)}$ over all possible programs that generate this artifact.

Another important work is Schmidhuber [53]’s attempt to formalize certain aspects of depicting essence of artistic objects. The author proposes that an artwork should “look right” and that its Kolmogorov complexity should be small; relating such properties to informal notions, such as “good artistic style” and “beauty”.

The technical discussions on the effectiveness of AIT in extracting meaningful information from low-entropy artifacts like music were pioneered by Cilibrasi et al. [54] and Cilibrasi and Vitanyi [55], where the authors approximate the Kolmogorov complexity K(x) of a symbolic music piece *x* with a general purpose compression scheme like LZ78. They defined a normalized information distance or

$$NID=\frac{K(xy)-\min\{K(x),K(y)\}}{\max\{K(x),K(y)\}},$$

between two music pieces *x* and *y*. The NID(x,y) is defined based on a measure of *x* and *y*’s shared information (the numerator), which is approximated using their combined compression length K(x,y).

If the two pieces are similar, then the compressor will leverage this redundancy and K(x,y) will be close to K(x) or K(y). Of course, general-purpose compressors may not be appropriate for this application. Mondol and Brown [9] demonstrated a practical method for approximating the conditional Kolmogorov complexity of a symbolic music string using a *context-free grammar* as a valid model for compressing the music sequences.

If two music pieces are similar, the context-free patterns of one can be used to compress a similar music piece. Ens and Pasquier [56] used NID to approximate the statistical significance of inter-corpora artifact distance between generated artifacts of a style imitation system and the reference artifacts, which it tries to emulate. Svangrd and Nordin [57] used the NID to predict how interesting new images would be to an observer comparing it to a library of aesthetic images.

In order to approximate the NID, they used a combination of ZIP and JPEG compression of the images and predict how “aesthetically pleasing” an artifact would seem to an observer by estimating its average NID from the images in the library. The authors then compare these predictions to an user’s evaluation of aesthetic quality of an image and show that the NID can distinguish between “ugly” and “pretty” images with an accuracy better than the random baseline. Their process of assessing “aesthetic pleasure” almost echoes McGregor’s attempt [25] to formalize *novelty*, we mentioned in Section 3.3.

The relationship between value and the computational effort of a source to generate an artifact was noticed through the formulation of *logical depth* [32]. Zenil et al. [12] used PNG decompression time as *logical depth* to show that images that we would normally consider valuable had a high decompression time (Section 4.2). Vidal and Delahaye [58], in particular, cited exactly this same quantity in their proposal of an ethical mandate to protect artifacts that contain computational significance of the same sort.

### A Formal Framework

We revisit the formulation of aesthetic beauty as the ratio $\frac{O}{C}$. Specifically, Rigau et al. [51] approximated this ratio with relative redundancy $\frac{\left|x|-K(x\right)}{\left|x\right|}$ equating order with |x|−K(x), the amount by which *x* can be compressed. While this might work for objects that lie at either extreme of randomness (strings formed by coin-toss or repetitions of bits), it fails precisely for objects that are in-between.

For a highly sophisticated object, this definition measures exactly the opposite of order, as such objects exhibit structure through almost all of their complexity. Kosheleva et al. [52] defines order in a similar manner with $2^{-l(p)}$, both arguing that the smaller l(p)=K(x), the more order $2^{-l(p)}$ or x−K(x) is there in the object. However, their definition of complexity with the runtime of the program, which witnesses the largest $2^{-l(p)}$, is potentially of concern.

We now know that time(p) is far from being a measure of complexity of an object; rather, it marks the derivation-time of an object from a plausible short program. Thus, a large time(p) will assign high complexity to an object that has high subjective organization and in fact, can be generated from a short program through a long computation.

We thus reformulate $\frac{O}{C}$ of an artifact, *x* by defining order with K(M), where *M* models the regularities in *x* leaving out the additional K(x|M) accidental information and K(M)+K(x|M)≤K(x)+O(1). Based on our discussion, *M* can be built either from a coherent set of inspiring objects with which we want to recognize *x* or it can be its sophistication or useful properties that can be used to generate similar objects.

The denominator or complexity is simply the raw Kolmogorov complexity K(x). Then, $\frac{K(M)}{K(x)}$ assigns highest aesthetic beauty to masterpieces and lowest to objects that exhibit low structure (random strings or sequences of 1’s have low complexity models: fair coin-toss generators or printing |x| 1’s).

Finally, we present the algorithmic recipe in Table 1, summarizing the concepts presented in this paper. This highlights an exciting mapping from creative entities to their counterparts in algorithmic information theory and can be followed for computational analysis of creativity and aesthetics.

## 7. Conclusions

We looked at some of the fundamental concepts of creativity and aesthetics through the lens of algorithmic information theory. We talked about the probability of an artifact’s existence on a universal machine and saw how Kolmogorov complexity favors artifacts with short descriptions, even though the overwhelming majority of objects in our universe are random and incompressible. We formalized the *typicality* and *novelty* of a never-before-seen artifact with a *model* and *data-to-model codes*, built from an inspiring set of already-observed objects.

Perhaps more importantly, we laid a groundwork for conceptualizing *value*. Historically, researchers have often attempted to perceive this dimension of an artifact with its compressibility. However, as was shown, we tend to assign value to artifacts, which cannot be produced easily and which display a high amount of subjective organization. We discussed what it means to be an authentic creative process: an observer or critic’s time-bounded explanation $K^{t}\left(x\right)$ of an artwork *x* can be influenced by the real creative process of the artist; while they can also dismiss the fraudulent claims of a charlatan by seeing through the actual value of an artifact.

Such interplay between artists and critics has been often absent from previous computational understandings of creative work and its quality. Additionally, the notion of *sophistication* lets us illustrate a creator’s virtuosity present in their creative product. The input *d* to a generator program *p* that we called accidental information, can be thought of as the inspiration or an encoding of the surrounding environment that influences a creator program *p*.

A particular delightful outcome of sophistication is its ability to describe masterpieces in the form of highly sophisticated artifacts, whose existence is only possible through an absolutely non-stochastic program. These concepts and formalization provide a reliable theoretical foundation upon which other models (e.g., machine learning) can be built and evaluated.

## Figures and Tables

**Table 1:** An algorithmic recipe for computational creativity and aesthetics.

Creative Entity	Attributes	Algorithmic Information Theory Notion
Artifact	Typicality	Randomness Deficiency
	Novelty	Mutual Information between model parameters
	Order and Noise	Model and data-to-model codes
Creative Process	Non-randomness	Logical Steps of *s*-significant program
	Value (also of artifact)	*s*-significant Logical Depth
Creator	Skills and Style	Sophistication
	Masterpiece	Non-stochasticity

## Data Availability

The data is contained within the article.

## Abbreviations

The following abbreviations are used in this manuscript:

AIT	Algorithmic Information Theory
